# Social Network Site Appearance Comparison's Prediction of Anxiety Among Chinese Females: The Mediation Effect of Body Area Satisfaction, Overweight Preoccupation, and Self-Esteem

**DOI:** 10.3389/fpsyt.2022.775782

**Published:** 2022-03-07

**Authors:** Ri Hai, Yin Yang

**Affiliations:** School of Psychology, Beijing Sport University, Beijing, China

**Keywords:** social network site appearance comparison, multidimensional body-self relations, self-esteem, anxiety, overweight preoccupation, body areas satisfaction

## Abstract

Social network site appearance comparison refers to a tendency to compare the body image of one-self to others when using social network sites. It was found to be associated with negative emotions, for example, depression, among young females, and this association was mediated by body image and self-esteem. However, researches on the chain-mediating role of body image and self-esteem in anxiety had been limited. Therefore, the current study examined the chain-mediating role between social network site comparison and anxiety among 320 Chinese females, using the Social Network Site Appearance Comparison Scale (SNSACS), Multidimensional Body–Self Relations Questionnaire (MBSRQ), Self-esteem Scale (SES), and the anxiety subscale of Depression-Anxiety-Stress Scale (DASS). Results revealed that (1) SNSACS, SES, and DASS anxiety scores were significantly correlated with each other, and the scores of two MBSRQ subscale, that is, body area satisfaction and overweight preoccupation, were significantly correlated with SNSACS, SES, and DASS anxiety scores; (2) body area satisfaction and self-esteem played a chain-mediating role in the effect of social network site appearance comparison on anxiety; (3) overweight preoccupation and self-esteem played a chain-mediating role in the effect of social network site appearance comparison on anxiety. The findings may inspire new ideas for understanding how social comparison triggers anxiety and for developing methods to reduce anxiety derived from appearance comparison among Chinese females.

## Introduction

In today's society, the demands on women's body shape are getting higher and higher. An ideal female body is considered to be not only slim, but also in perfect proportion. These high standards make the idea of beauty less healthy and natural, as more women are using weight-loss drugs or taking cosmetic surgery. Besides, women are becoming increasingly vulnerable to weight and appearance anxiety, while internet media makes the situation even worse. Women are surrounded by Internet media and inevitably affected by the appearance standards it promotes and make comparison inadvertently, which often provokes anxiety.

Social network site appearance comparison referred to the process in which an individual compares with other website users, features such as appearance and body shape through selfies, videos, and other information displayed on social network sites (1). Fardouly et al. (2) developed the Social Network Site Appearance Comparison Scale (SNSACS) to investigate the extent one compared his/her appearance, body shape, and clothing with others on social network sites. Social network site appearance comparison was associated with mental health. For instance, Fardouly et al. (3) found that making fewer appearance comparisons was positively associated with preadolescents' mental health. Social network site appearance comparison was also found to be positively associated with depression (4, 5).

Social network site appearance comparison was also associated with body image concerns. Body image was defined as people's psychological feelings toward their own bodies, including the perception, imagination, emotion, and physical properties (6) and was considered to be highly correlated with social network site appearance comparison. For instance, Baker et al. (7) found that female college students reported dissatisfaction toward their appearance and frequent comparison about their looks or the number of likes with others. Cohen et al. (8) further found that the engagement of photo activities, rather than the general use, in Facebook and Instagram led to thin-ideal internalization, body surveillance, and drive for thinness. Rousseau et al. (9) found that more appearance comparisons on Facebook were associated with more body dissatisfaction. Besides, women with higher comparison tendency were found to be more susceptible to the impact of mass media on their body image (10) and that the tendency of social network site appearance comparison was related to body shame, restrictive diet, and cognitive bias of self-image among female college students (11, 12). Appearance comparison in social network sites was also found to mediate the effect of Facebook usage on body image concerns and self-objectification (2, 13).

Body image concerns were also associated with self-esteem and anxiety. For instance, adults who were satisfied with their appearance and weight often had higher self-esteem and life satisfaction (14). Teenagers who had social media–related body dissatisfaction would check social media more frequently and have higher rates of depression and online social anxiety than those who did not (15). Both body image disturbance and low self-esteem were associated with higher level of social anxiety in adolescents (16–18). Besides, individual's recognition of body image was found to affect their anxiety about building intimate relationships, whereas self-esteem played a partial mediating role (19). Using the Multidimensional Body-Self Relations Questionnaire (MBSRQ) developed by Cash (20), Zhang et al. (21) found that only body area satisfaction and overweight preoccupation subscales of MBSRQ were associated with anxiety, and these associations were mediated by self-esteem.

Peng et al. (22) examined the chain-mediating effect of body image and self-esteem on the relationship between social network site appearance comparison and depression. In their study, appearance comparison was found to have a direct effect on depression, while it also affected depression indirectly, through the mediating effect of either body image satisfaction or self-esteem, respectively, and the chain-mediating effect of body image satisfaction and self-esteem (22). High tendency of appearance comparison could cause dissatisfaction on one's body image and then lower self-esteem and trigger depression.

While social network site appearance comparison was associated with depression (4, 5), its relationship with other negative emotions, for example, anxiety, remained unknown. Besides, results of previous studies (16–18, 21) indicated that body image, more specifically body area satisfaction and overweight preoccupation, and self-esteem may play mediating roles in the relationship between social network site appearance comparison and anxiety. Therefore, the current study aimed to investigate the relationships between social network site appearance comparison, multidimensional body-self relations, self-esteem, and anxiety among a sample of Chinese females, and the hypotheses were as follows:

(1) Social network site appearance comparison, body area satisfaction and overweight preoccupation subscales of MBSRQ, self-esteem, and anxiety were correlated with each other.

(2) Body area satisfaction and self-esteem had a chain-mediating effect between social network site appearance comparison and anxiety.

(3) Overweight preoccupation and self-esteem had a chain-mediating effect between social network site appearance comparison and anxiety.

## Materials and Methods

### Participants

A total of 359 female college students or graduates were recruited from an online survey platform (https://www.wjx.cn/jq/92808961.aspx), whereas 39 of them were excluded because of the following reasons: (1) 18 participants were excluded because they were male; (2) 7 participants were excluded because their answering times were <400 s; (3) 9 participants were excluded because they failed to respond correctly to one or more attention check question(s); and (4) 5 participants were excluded because they were older than 40 years. Therefore, 320 female participants were ultimately retained, whose ages ranged from 17 to 39 years, with a mean age of 21.6 ± 3.2 years.

### Measurements

#### Social Network Site Appearance Comparison Scale

SNSACS, developed by Fardouly et al. (2), is a three-item scale measuring the extent one would compare his/her appearance, body shape, and clothing with others when using social network sites. The SNSACS used a 5-point Likert scale, where 1 refers to “strongly disagree” and 5 “strongly agree.” Higher scores indicate that one was more likely to compare with others when using social networking sites. The Chinese version of SNSACS was translated by Peng (23) and had good reliability in the current study (item-total correlation = 0.879–0.911, Cronbach α = 0.879).

#### Multidimensional Body–Self Relations Questionnaire

The MBSRQ was developed by Cash (20) to measure one's attitudes toward his/her body image. The original questionnaire consisted of 69 items, using a 5-point Likert scale, where 1 refers to “definitely disagree,” and 5 refers to “definitely agree.” The MBSRQ measured 10 dimensions of body image, which were appearance evaluation (APPEVAL), appearance orientation (APPOR), health evaluation (HLTHEVAL), health orientation (HLTHOR), fitness evaluation (FITEVAL), fitness orientation (FITOR), illness orientation (ILLOR), Body Areas Satisfaction Scale (BASS), overweight preoccupation (OWPREOC), and self-classified weight (WTCLASS), respectively. Ma (24) translated and revised the Chinese version of MBSRQ, which retained the same 10 subscales but consisted of 93 items. The Chinese version of MBSRQ had fine internal consistency in its eight subscales (Cronbach α = 0.57–0.84), except for that in the FITEVAL (Cronbach α = 0.28) and HLTHOR (Cronbach α = 0.40) subscales. Besides, the test–retest reliability of the Chinese version of MBSRQ was 0.665 (24).

#### Rosenberg Self-Esteem Scale

The Rosenberg Self-esteem Scale (SES) (25) consisted of 10 items and evaluated one's overall attitudes toward his/her worthiness as a human being (26). The responses were recorded on a 4-point scale, ranging from 1 (strongly disagree) to 4 (strongly agree). The Chinese version of SES was translated and revised by Ji and Yu (27), with the eighth item rewritten (28). Higher scores indicate a higher level of self-esteem. The Cronbach α of the SES in the current study was 0.902.

#### The Anxiety Subscale of Depression-Anxiety-Stress Scale-21

The Depression-Anxiety-Stress Inventory-21 (DASS-21) was developed by Lovibond and Lovibond (29), which consisted of three subscales: depression, anxiety, and stress. In the current study, only the anxiety subscale was used. The anxiety subscale consisted of seven items, which were rated on a 4-point scale ranging from 0 (never) to 3 (almost always). Higher scores indicate more severe anxiety symptoms (30). The simplified Chinese version of DASS-21 was revised by Gong et al. (31), and the Cronbach α of the anxiety subscale was 0.80.

### Procedure and Data Analysis

The questionnaire was delivered online with a QR code through a social app named WeChat; once the participants scanned the QR code, they would jump to the web page of this questionnaire. The participants would carefully read the informed consent written by the researcher; only if they agreed with it could they go on to read the instructions written by the researcher in the questionnaire and fill the questionnaires in according to it. After filling in the questionnaires, all participants would receive 3 RMB as remuneration through WeChat in 3days.

Correlation analysis and chain-mediated analysis were performed on the collected data using SPSS 25.0 and the PROCESS macro for SPSS (version 2.16.3) developed by Hayes (32). The number of bootstrap samples was set at 5,000, and the confidence interval was set at 95%.

## Results

### Correlations Among SNSAC, MBSR, Self-esteem, and Anxiety

Pairwise correlation analysis revealed the following: (1) social network site appearance comparison, self-esteem, and anxiety scores were significantly correlated with each other (*r* = −0.138, 0.140, and −0.413, respectively; all *p* < 0.05); (2) social network site appearance comparison was also significantly correlated with appearance orientation (*r* = 0.471, *p* < 0.01), body area satisfaction (*r* = −0.141, *p* < 0.05), and overweight preoccupation (*r* = 0.304, *p* < 0.01) subscale scores of MBSRQ; (3) except for appearance orientation and self-classified weigh subscales, scores of the other eight subscales of MBSRQ were significantly correlated with both self-esteem (*r* = −0.144 to 0.505, all *p* < 0.01) and anxiety (*r* = −0.274 to 0.168; all *p* < 0.01 except for that of ILLOR subscale, *p* < 0.05) scores. See detailed descriptive statistics and correlation coefficients among all measures in Table 1.

**Table 1:** Descriptive statistics of measures and their correlation coefficients.

	**Mean**	**SD**	**SNSACS**	**SES**	**Anxiety**
**SNSACS**	9.98	3.05			
**SES**	30.28	5.42	−0.138*		
**Anxiety**	12.62	8.71	0.140*	−0.413**	
**MBSRQ**
Appearance evaluation	3.27	0.69	−0.079	0.491**	−0.274**
Appearance orientation	3.39	0.49	0.471**	−0.023	0.064
Fitness evaluation	3.16	0.9	−0.018	0.302**	−0.167**
Fitness orientation	3.41	0.65	−0.091	0.300**	−0.240**
Health evaluation	3.61	0.7	−0.04	0.381**	−0.421**
Health orientation	3.33	0.5	0.021	0.259**	−0.190**
Illness orientation	3.6	0.64	−0.003	0.292**	−0.131*
Body area satisfaction scale	3.24	0.75	−0.141*	0.505**	−0.256**
Overweight preoccupation	2.85	0.9	0.304**	−0.144**	0.168**
Self-classified weight	2.74	0.5	0.037	−0.063	0.052

* *p < 0.05*,

** *p < 0.01*.

*SNSACS, social network site appearance comparison scale; SES, self-esteem scale; MBSRQ, multidimensional body–self relations questionnaire*.

### The Chain-Mediating Effect Analysis

The MBSRQ includes 10 dimensions, so there were a total of 10 underlying models that need to be analyzed. Because the premise of implementing mediating effect analysis was that there was correlativity among predictors (i.e., independent variables), mediating variables, and criteria (i.e., dependent variables), only 2 models met the correlativity in all pathways, which were as follows: (1) social network site appearance comparison was taken as the predictor, body area satisfaction and self-esteem were taken as the mediating variables, and anxiety was taken as the criterion, named as model 1 and (2) social network site appearance comparison was taken as the predictor, overweight preoccupation and self-esteem were taken as mediating variables, and anxiety was taken as the criterion, named as model 2.

**Figure 1:**
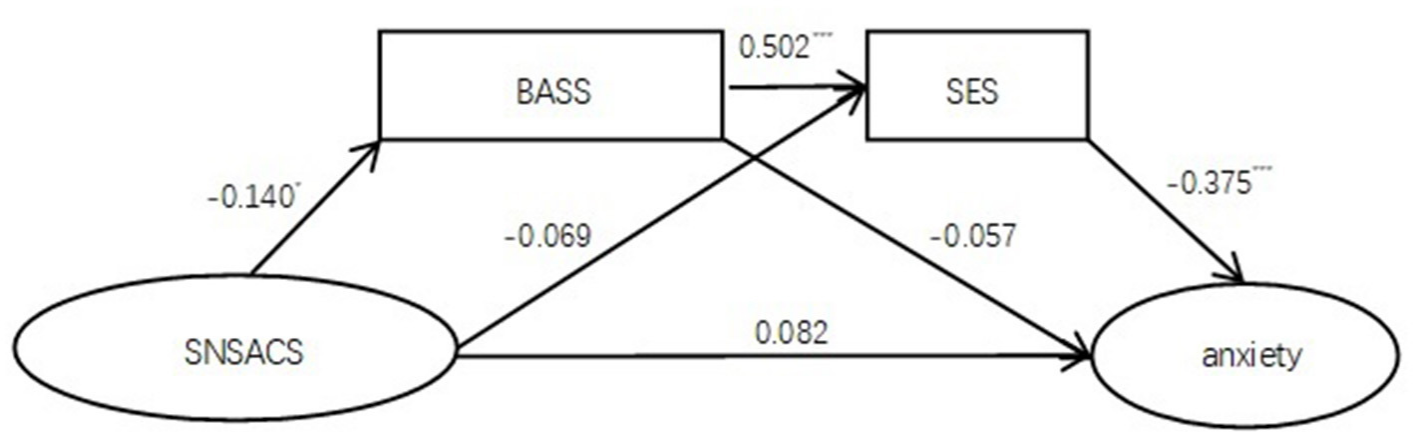
The BASS and SES significantly played a chain mediating effect between SNSACS and anxiety. SNSACS, social network site appearance comparison scale; BASS, body areas satisfaction scale; SES, self-esteem scale. *p < 0.05, ***p< 0.001.

The chain-mediating effect analysis of model 1 revealed the following: (1) the overall model was significant (*R*^2^ = 0.180, *F* =23.147, *p* < 0.001); (2) the direct effect between social network site appearance comparison and anxiety failed to reach a significant level (95% CI = −0.021 to 0.184), whereas the total mediating effect was significant (95% CI = 0.017–0.114); (3) social network site appearance comparison negatively predicted body area satisfaction (β = −0.140, *t* = −2.532, *p* = 0.012), body area satisfaction positively predicted self-esteem (β = 0.502, *t* = 10.143, *p* < 0.001), and self-esteem negatively predicted anxiety (β = −0.375, *t* = −6.320, *p* < 0.001); (4) the chain-mediating effect was significant (95% CI = 0.006–0.057). The results of chain-mediating effect analysis are shown in Table 2 and Figure 1.

**Table 2:** The mediating effects and proportions of chain-mediating models.

**Model**	**Direct/indirect effects**	**Effect size**	**Proportion**	**Boot LLCI**	**Boot ULCI**
Model 1: The chain-mediating model of SNSAC → BASS → self-esteem → anxiety	Direct effect	0.082		−0.021	0.184
	Total mediating effect	0.060	29.70%	0.017	0.114
	SNSAC → BASS → anxiety	0.008	3.96%	−0.007	0.037
	Chain-mediating effect	0.026	12.87%	0.006	0.057
	SNSAC → self-esteem → anxiety	0.026	12.87%	−0.011	0.070
	Total effect	0.202			
Model 2: The chain-mediating model of SNSAC → OWPREOC → self-esteem → anxiety	Direct effect	0.058		−0.049	0.165
	Total mediating effect	0.084	37.17%	0.034	0.147
	SNSAC → OWPREOC → anxiety	0.029	12.83%	0.001	0.068
	Chain-mediating effect	0.014	6.19%	0.0003	0.032
	SNSAC → self-esteem → anxiety	0.041	18.14%	0.001	0.093
	Total effect	0.226			

*SNSAC, Social Network Site Appearance Comparison; BASS, Body Areas Satisfaction Scale; OWPREOC, overweight preoccupation; LLCI, lower level of confidence interval; ULCI, upper level of confidence interval*.

**Figure 2:**
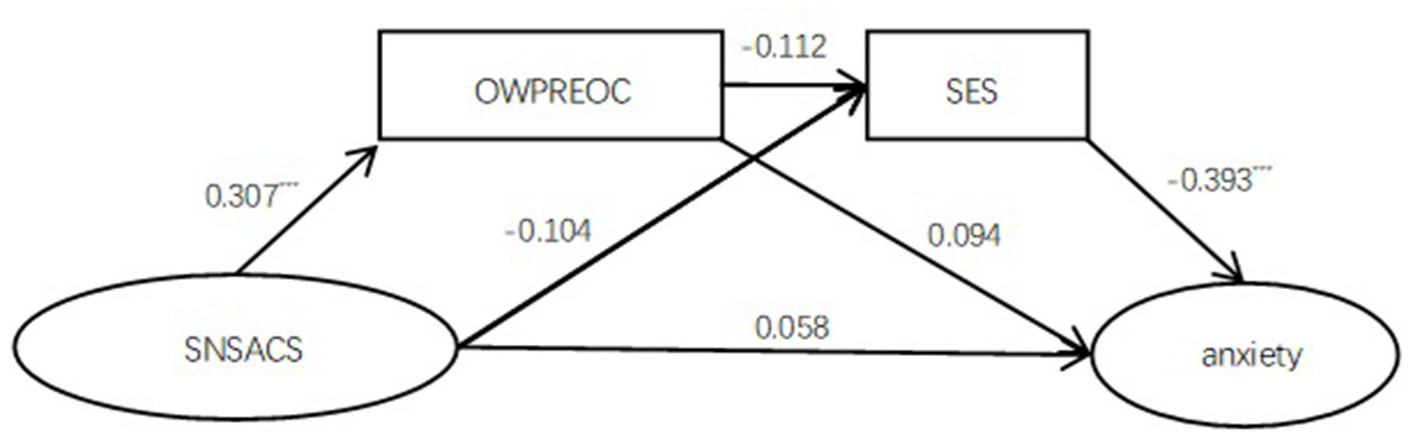
The OWPREOC and SES significantly played a chain mediating effect between SNSACS and anxiety. Besides, OWPREOC and SES were found to have significant indirect effect, respectively. SNSACS, social network site appearance comparison scale; OWPREOC, overweight preoccupation; SES, self-esteem scale. ***p < 0.001.

The chain-mediating effect analysis of model 2 revealed that (1) the overall model was significant (*R*^2^ = 0.186, *F* =24.042, *p* < 0.001); (2) the direct effect social network site appearance comparison effect on anxiety failed to reach a significant level (95% CI = −0.049 to 0.165), whereas the total mediating effect was significant (95% CI = 0.034–0.147); (3) social network site appearance comparison positively predicted overweight preoccupation (β = 0.307, *t* = 5.694, *p* < 0.001), overweight preoccupation marginally predicted self-esteem (β = −0.112, *t* = −1.936, *p* = 0.054), and self-esteem negatively predicted anxiety (β = −0.393, *t* = −7.601, *p* < 0.001); (4) the effect of social network site appearance comparison on anxiety was mediated by overweight preoccupation (95% CI = 0.001–0.068) and self-esteem (95% CI = 0.001–0.093), respectively; the chain-mediating effect was significant (95% CI = 0.0003–0.0324). The results of chain-mediating effect analysis are shown in Table 2 and Figure 2.

## Discussion

In the current study, we examined the relationships between social network site appearance comparison, multidimensional body-self relations, self-esteem, and anxiety among a sample of Chinese female young adults. We found that social network site appearance comparison had significant positive correlations with anxiety. Moreover, the body area satisfaction and overweight preoccupation dimensions of MBSRQ and self-esteem were found to have a chain-mediating effect on the relationship between social network site appearance comparison and anxiety. Previous studies had revealed the effect of social network site appearance comparison on depression (4, 5) and the mediating role of body image satisfaction and self-esteem (22). The current study extended our understanding of the effect of social network site appearance comparison on anxiety and revealed chain-mediating effects of body image and self-esteem, which was consistent with previous studies (22). More particularly, by using a multidimensional measure of body image (i.e., MBSRQ), we found that two dimensions of body image, that is, body area satisfaction and overweight preoccupation, were most relevant to social network site appearance comparison, self-esteem, and anxiety. These two dimensions had been found to be associated with self-esteem and anxiety (21). These results contributed to our understanding of how different dimensions of body image vary on their associations with appearance comparison and anxiety. Comparing one's appearance on social network site among young females would provoke more anxiety, through lowering one's satisfaction with her own body parts and inducing more concerns about being overweight, which then lowered one's self-esteem.

The results of the current study may also have clinical implications. For instance, the results of the current study indicate that body area satisfaction and overweight preoccupation might have more to do in the relationship between social network site appearance comparison and anxiety, compared with other dimensions of body-self relations. These results may provide a preliminary empirical support for intervention target for the emotional problems caused by body image comparison. Future researches could further explore how to effectively intervene on appearance satisfaction, overweight preoccupation, and self-esteem, so as to achieve the purpose of promoting the emotional health of females.

The current study had several limitations. First, the researcher made a mistake and failed to collect data of item 18 of the appearance orientation subscale in the MBSRQ. This mistake might have an impact on the results of the current study; the interpretation of the results should also be treated with caution. Second, this study is a cross-sectional study; thus, caution should be taken when making inferences about causality between variables. Future studies can use the longitudinal study design method to explore the causal relationship between variables. Third, we mainly recruited female young adults in the current study. It should be noted that women of different ages may vary in social media use and in its impact on anxiety. Therefore, one should be cautious when generalizing the results of the current study to women of other age ranges.

## Data Availability Statement

The raw data supporting the conclusions of this article will be made available by the authors, without undue reservation.

## Ethics Statement

Ethical review and approval was not required for the study on human participants in accordance with the local legislation and institutional requirements. Written informed consent for participation was not required for this study in accordance with the national legislation and the institutional requirements.

## Author Contributions

RH and YY conceived the study and drafted the paper. RH collected and analyzed the data. Both authors contributed to the article and approved the submitted version.

## Conflict of Interest

The authors declare that the research was conducted in the absence of any commercial or financial relationships that could be construed as a potential conflict of interest.

## Publisher's Note

All claims expressed in this article are solely those of the authors and do not necessarily represent those of their affiliated organizations, or those of the publisher, the editors and the reviewers. Any product that may be evaluated in this article, or claim that may be made by its manufacturer, is not guaranteed or endorsed by the publisher.
